# Promoting Roles of Embryonic Signals in Embryo Implantation and Placentation in Cooperation with Endocrine and Immune Systems

**DOI:** 10.3390/ijms21051885

**Published:** 2020-03-10

**Authors:** Hiroshi Fujiwara, Masanori Ono, Yukiyasu Sato, Kazuhiko Imakawa, Takashi Iizuka, Kyosuke Kagami, Tomoko Fujiwara, Akihito Horie, Hirohiko Tani, Akira Hattori, Takiko Daikoku, Yoshihiko Araki

**Affiliations:** 1 Department of Obstetrics and Gynecology, Kanazawa University Graduate School of Medical Science, Kanazawa 920-8641, Japan; masanori@med.kanazawa-u.ac.jp (M.O.); zukatti713@gmail.com (T.I.); ktykkn@yahoo.co.jp (K.K.); 2 Department of Obstetrics and Gynecology, Takamatsu Red Cross Hospital, Takamatsu 760-0017, Japan; yukiyasu@kuhp.kyoto-u.ac.jp; 3 Research Institute of Agriculture, Tokai University, Kumamoto 862-8652, Japan; ik459102@tsc.u-tokai.ac.jp; 4 Department of Home Science and Welfare, Kyoto Notre Dame University, Kyoto 606-0847, Japan; fujiwara@notredame.ac.jp; 5 Department of Obstetrics and Gynecology, Kyoto University Graduate School of Medicine, Kyoto 606-8507, Japan; a_horie@kuhp.kyoto-u.ac.j (A.H.); tanita@kuhp.kyoto-u.ac.jp (H.T.); 6 Department of System Chemotherapy and Molecular Sciences, Kyoto University Graduate School of Pharmaceutical Sciences, Kyoto 606-8501, Japan; ahattori@pharm.kyoto-u.ac.jp; 7 Division of Transgenic Animal Science, Advanced Science Research Center, Kanazawa University, Kanazawa 920-8640, Japan; tdaikoku@kiea.m.kanazawa-u.ac.jp; 8 Institute for Environmental and Gender-specific Medicine, Juntendo University Graduate School of Medicine, Urayasu 279-0021, Japan; yaraki@juntendo.ac.jp; 9 Department of Obstetrics and Gynecology, Juntendo University Graduate School of Medicine, Tokyo 113-8421, Japan

**Keywords:** embryo implantation, embryonic signal, immune system, immune therapy, implantation failure, placentation

## Abstract

Embryo implantation in the uterus is an essential process for successful pregnancy in mammals. In general, the endocrine system induces sufficient embryo receptivity in the endometrium, where adhesion-promoting molecules increase and adhesion-inhibitory molecules decrease. Although the precise mechanisms remain unknown, it is widely accepted that maternal–embryo communications, including embryonic signals, improve the receptive ability of the sex steroid hormone-primed endometrium. The embryo may utilize repulsive forces produced by an Eph–ephrin system for its timely attachment to and subsequent invasion through the endometrial epithelial layer. Importantly, the embryonic signals are considered to act on maternal immune cells to induce immune tolerance. They also elicit local inflammation that promotes endometrial differentiation and maternal tissue remodeling during embryo implantation and placentation. Additional clarification of the immune control mechanisms by embryonic signals, such as human chorionic gonadotropin, pre-implantation factor, zona pellucida degradation products, and laeverin, will aid in the further development of immunotherapy to minimize implantation failure in the future.

## 1. Main Factors Regulating Endometrial Receptivity for Embryo Implantation

### 1.1. Endocrine System and Embryo Signals

In mammals, embryo implantation in the uterus is an essential process in successful pregnancy. In general, the endocrine system regulates endometrial differentiation such that the embryo can be implanted. The initial endometrial differentiation is induced by estrogen. Then, progesterone stimulates this estrogen-primed endometrium to differentiate further to make it more suitable for embryo implantation [1,2]. Estrogen is mainly secreted from growing follicles and progesterone is produced by the corpus luteum, a newly formed endocrine organ originating from the ovulated follicle. This sequential endocrine stimulation is closely coordinated with the estrus cycle, creating the endometrial receptive phase, referred to as an implantation window [3,4,5,6].

It is widely believed that such an implantation window, which spans from a few days after ovulation to several days prior to menstruation, also exists in women [7]. The human implantation window is estimated to correspond to cycle days 20 to 24 of the menstrual cycle [8]. However, there has been no study to directly confirm this window. Therefore, we developed an attachment assay using a human choriocarcinoma cell line, BeWo cells, and human primary endometrial epithelial cell culture to examine whether human endometrial receptivity changes during the menstrual cycle [9]. In this assay, high attachment rates were observed in endometrial culture derived from the mid-luteal phase. Of note, except for the mid-luteal phase, the attachment rates were low, suggesting that human endometrial receptivity changes during the menstrual cycle [10]. As BeWo cells easily attached to endometrial stromal cells or human endometrial carcinoma-derived Ishikawa cells, we suggest that certain adhesion-inhibitory factors are present on the endometrial epithelial cell layer. Consequently, we hypothesized that in the receptive phase, adhesion-promoting molecules increase, whereas adhesion-inhibitory molecules decrease. In addition, structural changes of epithelial cells, the so-called uterodome, were reported to be induced on the cell surface of luminal epithelial cells, which are suggested to be involved in embryo attachment to the endometrial epithelial layer [11,12].

Concomitant with hormonal preparation, direct cross-talk between the embryo and maternal endometrium is considered necessary to achieve subsequent successful embryo implantation [13,14]. The blastocyst will implant only when this molecular cross-talk is established [15,16]. Although the precise mechanisms remain unknown, it was proposed that human chorionic gonadotropin (hCG) is one of the important embryonic signals that increases the receptive ability of the sex steroid hormone-primed endometrium [17,18]. In nonhuman primates, hCG directly induced the expression of α-smooth muscle actin (SMA) in baboon endometrial stromal cells and glycodelin in the glandular epithelium, suggesting that the primate blastocyst signal alters the uterine environment prior to implantation [19]. In humans, the intrauterine administration of hCG using an intrauterine microdialysis system was reported to inhibit the expression of differentiation parameters: insulin-like growth factor binding protein-1 (IGFBP-1) and prolactin, while increasing the expression of implantation-related factors; leukemia inhibitory factor (LIF) and macrophage colony stimulating factor (M-CSF), and a neoangiogenetic factor: vascular endothelial growth factor (VEGF), in the mid-luteal human endometrium, suggesting that hCG regulates endometrial differentiation and vascularization [20,21]. Recently, hyperglycosylated hCG, an hCG isotype with larger N- and O-linked oligosaccharides, was suggested to play an important role in embryo implantation [22,23].

In addition to soluble factors, microRNAs secreted from human blastocysts were proposed to be new embryonic signals that regulate adhesive properties of endometrial epithelial cells. miR-661 from nonimplanted human blastocysts was taken up by primary human endometrial epithelial cells and it reduced the attachment of trophoblast cell line spheroids to these epithelial cells [24]. Later, the role of other noncoding RNAs in maternal–embryo communication through extracellular vesicles was observed, demonstrating the noncontact transfer of embryonic RNA transcripts to the endometrium and the altered expression of endogenous transcripts by endometrial cells [25]. A recent study also proposed that an embryo-secreted long noncoding RNA, phosphatase and tensin homolog pseudogene 1 (*PTENP1*), is involved in the endometrial adhesive properties [26].

### 1.2. Adhesion-Promoting and -Inhibiting Molecules

For human embryo implantation, several adhesion-promoting molecules, such as trophinin [27,28,29], L-selectin ligand [30,31,32,33], integrin αVβIII [34,35,36,37], and CD44 [38,39,40], were demonstrated to be expressed on human endometrial epithelial cells during the receptive phase. The expression of integrin βIII was found to be promoted by the embryonic interleukin (IL)-1 system, supporting the presence of cross-talk between blastocysts and the endometrial epithelium during embryonic implantation [41]. Recently, integrin αVβ3 and αVβ5 were reported to be necessary for the leukemia inhibitory factor-mediated adhesion of trophoblast cells to endometrial cells [36], whereas cell-surface CD44-hyaluronate binding was proposed to be employed by embryos during initial docking to endometrial epithelial cells [40]. This molecule is also involved in trophoblast invasion [42]. Consistent with the above report, we reported the possibility that versican, a large chondroitin sulfate proteoglycan that binds to hyaluronan and forms large extracellular matrix (ECM) aggregates, promotes human embryo attachment to endometrial epithelial cells [43]. In the bovine uterus, we also proposed that the interaction between vascular cell adhesion molecule 1 (VCAM1) in endometrial luminal cells and integrin α4 expressed on the conceptus functions in conceptus adhesion to the uterine endometrium [44].

As a regulatory cell surface protein of adhesion-promoting molecules, we previously reported that CD9, which regulates integrin function, is specifically expressed on the endometrial luminal and glandular epithelial cells [45]. CD9 and CD98 were subsequently demonstrated to function in embryo implantation [46,47,48]. CD9 is also involved in trophoblast invasion [49,50,51].

On the other hand, large glycoproteins, such as MUC1, that inhibit the physiological cell-to-cell interaction were expressed on murine and human luminal epithelial layers [52,53]. Later, the expression of MUC1 was found to be downregulated by human blastocysts using in vitro experiments, suggesting that MUC1 acts as an adhesion-inhibiting molecule that is locally removed by human blastocysts during the adhesion phase [54]. Another large glycoprotein, MUC16, was also found to be expressed on human endometrial epithelial cells, demonstrating that its expression is reduced on the cell surface of epithelial cells comprising the uterodome [55].

The above findings suggest that the expression of adhesion-promoting molecules increases, whereas that of adhesion-inhibiting molecules decreases according to embryonic signals during an implantation window.

### 1.3. Repulsive Molecules

**Figure 1:**
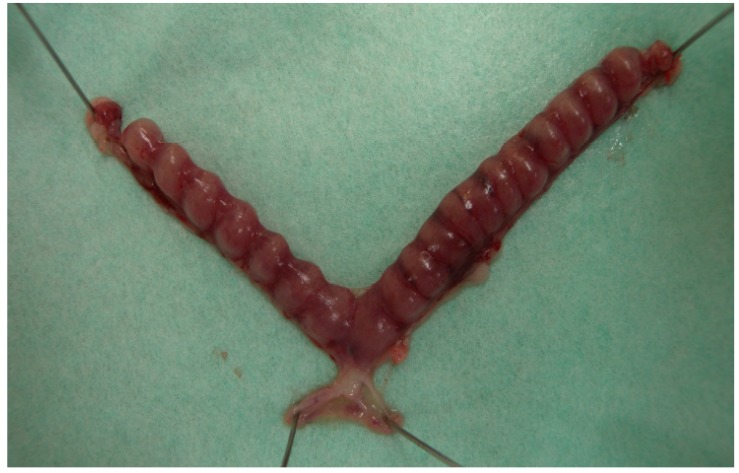
Equidistance of implantation sites in the murine uterus. In the pregnant murine uterus, the implantation sites are regulated equidistantly in response to numbers of implanting embryos.

A sufficient period is necessary for the cross-talk between embryo and mother in order to regulate embryo attachment to the endometrium with accurate timing and placement [56]. For example, implantation sites are regulated equidistantly in response to the number of implanting embryos, as observed in the pregnant murine uterus (Figure 1). However, it is difficult to explain the precise mechanisms leading to equidistance of implantation sites only by the balance of expression profiles between adhesion-promoting and adhesion-inhibiting molecules. Previously, Chen et al. excellently described the possible mechanisms determining the sites of embryo implantation, proposing factors such as the uterine recognition of embryos, fine-tuned uterine peristaltic movements, time-controlled uterine fluid reabsorption and uterine luminal closure, and embryo orientation [57]. To achieve the above sequential events, we should focus on alternative mechanisms that prevent embryos from immediately attaching to an inappropriate site of the endometrium.

From hatching to attachment, the bovine embryo floats in the uterine cavity for 4–5 days and becomes elongated prior to attachment [58,59,60]. This period may be an essential stage for embryo–maternal cross-talk. However, there is little information concerning molecules that assure sufficient distances between the embryo and endometrium before attachment. In mice, ovariectomy before pre-implantation on day 4 of pregnancy induces delayed implantation under progesterone supplementation [61]. When estrogen is administered, dormant blastocysts become activated and initiate implantation in the progesterone-primed uterus. During this blastocyst–uterine crosstalk before implantation, the endometrium undergoes further differentiation, secreting LIF. Notably, heparin-binding epidermal growth factor-like signaling was found to be induced in the activated blastocysts [62].

To investigate the mechanism to prevent premature embryo attachment, we hypothesized the involvement of repulsive forces. As repulsive force-inducing molecules, we observed that human maternal endometrial epithelial cells express ephrin A1 and human blastocysts express Eph A1, leading to the proposal that the Eph–ephrin system is involved in the initial interaction between human blastocysts (Eph A1) and the endometrium (ephrin A1) [63]. Ephrin A is a ligand for Eph A, and both molecules are located on the cell surface [64,65]. The ligand–receptor binding induces bidirectional signals affecting both cells, alters the functions of adhesion molecules [66], and promotes cell-to-cell adhesion, inducing arterio-venous anastomosis [67,68]. Of note, it also produces repulsive forces between the cells to induce axonal guidance [69].

Based on this background, we examined the time-course of mRNA expression of Eph/ephrin A in murine embryos. All subtypes of ephrin A were expressed on blastocysts. Importantly, around the hatching period, ephrin A expression transiently decreased and then increased again before attachment. Immunostaining revealed that ephrin A1 and A3 were expressed on the cell surface of the trophectoderm of blastocysts. Moreover, ephrin A3 expression on the blastocysts was weak on the opposite side of the inner cell mass, suggesting that the Eph–ephrin system acts as a regulator of the apposition site [70].

**Figure 2:**
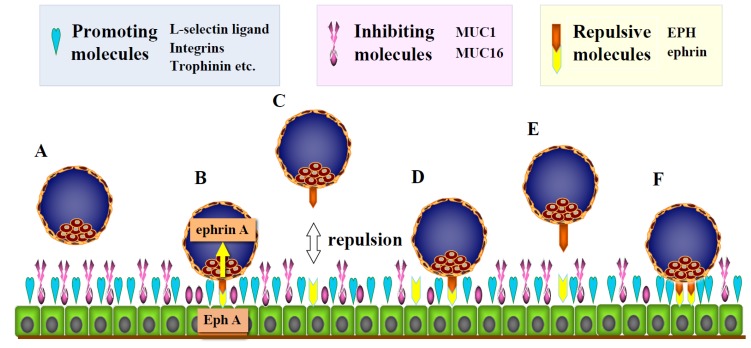
Possible mechanisms to create an adequate period of cross-talk between the embryo and mother before attachment by repulsive forces. (**A**) During human embryo implantation, several adhesion-promoting molecules, such as trophinin, L-selectin ligand, and integrin αVβIII, are expressed on endometrial epithelial cells during the receptive phase. In contrast, large glycoproteins, such as MUC1, that inhibit the physiological cell-to-cell interaction are expressed on the murine and human luminal epithelial layer. (**B**) During the implantation window, the expression of adhesion-promoting molecules increases, whereas that of adhesion-inhibiting molecules decreases in cooperation with embryonic signals, leading to cell-to-cell interaction between the embryo and endometrial epithelial cells. This enables the embryo to receive Eph signals from endometrial epithelial cells through ephrin ligands on its surface, leading to repulsive forces between the embryo and endometrium. (**C**–**E**) By repulsive forces through the Eph–ephrin system, the embryo separates from the endometrium (white two-way arrow), repeating attachment and detachment. (**F)** Finally, the embryo attaches to appropriate sites with suitable timing.

On the other hand, Eph A1, which is a receptor of ephrin A, was expressed on the murine endometrial luminal epithelial cells before embryo attachment. After attachment, EphA1 expression markedly decreased at the implantation site, whereas its expression on the luminal epithelial cells was maintained at nonimplanted sites [70]. Although embryo attachment was inhibited on Eph A1-coated dishes, when floating blastocysts were transferred to the control dishes, they gradually attached and spread on the dishes, suggesting that the Eph A signal induces repulsive forces [70]. Considering these findings, we propose that the Eph–ephrin system functions in creating a sufficient period for cross-talk between the embryo and mother, providing embryos with an opportunity to attach to appropriate sites by repeating attachment and detachment in a timely manner (Figure 2). Consistent with the above suggestion, the Eph–Ephrin A system was also reported to regulate the contact between blastocysts and endometrium during embryo implantation in swine [71,72], and low expression of Eph–ephrin A1 was later demonstrated to be related to an increase in the number of embryos during implantation [73].

## 2. Invasion Process of Human Embryos after Attachment to Endometrial Epithelial Cells

### 2.1. Activation of the Trophectoderm after Attachment and Acquisition of Invasive Properties

After attachment, the human embryo initiates intraepithelial invasion. Activation of the trophectoderm is considered to be an essential process for subsequent embryo invasion through intraepithelial spaces toward the endometrial stroma [27]. The trophectoderm layer at the endometrial site continues to be activated during the migrating process of human embryos on day 7.5 after ovulation. However, the precise mechanisms leading to this activation remain unknown. As candidates for activation-inducing molecules, Sugihara reported that trophinin is expressed at the interacting site between the embryo and endometrium and proposed that trophinin-mediated cell adhesion functions as a molecular switch for trophectoderm activation in human embryo implantation through trophinin-dependent tyrosine phosphorylation [27].

As other candidates, we reported that activated leukocyte cell adhesion molecule (ALCAM) is expressed on both human blastocysts and endometrial epithelial cells [74]. ALCAM is a transmembrane molecule that belongs to the immunoglobulin superfamily and mediates cell-to-cell adhesion by ALCAM–ALCAM homophilic interactions or ALCAM–CD6 heterophilic interactions [75]. By oligomerization, conjugated ALCAM molecules further form a cluster, increasing their adhesive ability [76]. Furthermore, ALCAM–ALCAM interaction induces the differentiation of stem cells. In immune cells, CD9 directly associates with ALCAM, regulating homophilic (ALCAM–ALCAM) and heterophilic (ALCAM–CD6) interactions, increasing ALCAM-mediated cell adhesion and T-cell migration, activation, and proliferation [77]. ALCAM was detected on the cell surface of human endometrial glandular and luminal epithelial cells by immunohistochemical staining and flow cytometry, whereas ALCAM expression on the human embryo was detected in the blastocyst stage by immunostaining, suggesting that ALCAM–ALCAM interaction is associated with human embryo attachment to the endometrium [74]. However, several proposed cell-adhesion molecules, including ALCAM, were not detected by DNA microarray analysis of human trophectoderm biopsy samples [78]. This should be clarified using current single-cell transcriptome analyzing systems.

### 2.2. Opening of Tight Junctions in the Endometrial Epithelial Layer

After attachment, the activated embryo initiates intraepithelial invasion in the next step and invades the maternal endometrial stromal tissues as a mass, opening the epithelial cell layer. During this process, the intercellular connection of the endometrial epithelial layer becomes reduced, maintaining the connection between the embryo-attached epithelial cells and basement membrane.

In general, the epithelial layer is tightly conjugated as a barrier. Notably, immune cells migrate through the epithelial layer from the basolateral to apical membrane [79,80]. Adhesion molecules, such as CD11b/CD18 and CD47, were reported to be necessary for sequential opening of the epithelial layer [81,82]. Dendritic cells were reported to open a lateral connection of epithelial cells, recruiting bacterial antigens from the mucosal epithelium to lymphoid tissues [83]. Although the precise mechanisms are still unclear, dendritic cells were also reported to express tight-junction proteins such as occludin, claudin 1, and zonula occludens 1 [83].

During human embryo invasion, the activated trophectoderm migrates to the intercellular spaces of epithelial cells with reduced tight junctions. Through this process of trophoblast invasion, the connection between epithelial cells and the basement membrane must be maintained. Proteases degrade the epithelial structures to weaken the tight junctions among epithelial cells. However, they also reduce attachment to the basement membrane, causing detachment of the epithelial cell layer that maintains embryo attachment. In contrast to the immune system, epithelial–mesenchymal transition (EMT) was reported to be involved in opening of the epithelial layer [84,85,86,87]. However, EMT simultaneously reduces the connection of epithelial cells to the basement membrane, undermining the anchoring foundation of the implanting embryo. Consequently, EMT is not suitable for subsequent human trophoblast invasion.

**Figure 3:**
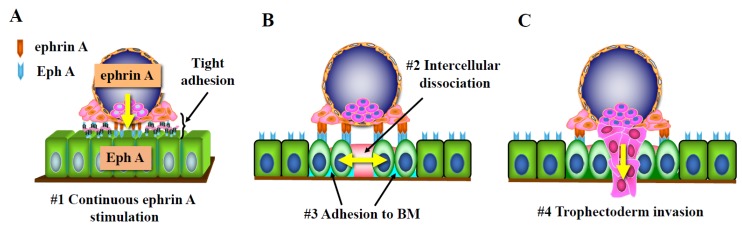
Possible mechanisms of human embryo invasion after attachment to endometrial epithelial cells. (**A**) After the embryo has firmly attached to the endometrium via several adhesion molecules, luminal epithelial cells receive continuous ephrin A signaling from the attached embryo (#1). (**B**) The Eph–ephrin system induces reduction of the tight junction of epithelial cells (#2), opening the epithelial cell layer (yellow two-way arrow). This system also promotes cell attachment to the extracellular matrix (#3) without destroying the connection between the embryo-attached epithelial cells and the basement membrane (BM). (**C**) This system may promote migration of the activated trophectoderm (#4) into the intercellular spaces of epithelial cells with reduced tight junctions.

After the embryo has firmly attached to the endometrium via several adhesion molecules, luminal epithelial cells receive continuous signaling from the attached embryo. The epidermal growth factor (EGF)-receptor system was proposed to induce the reduction of tight junctions of luminal epithelial cells by reducing the local calcium concentration at the trophoblast–endometrial interface [88]. As other candidates, we paid attention to the Eph–ephrin A system again because after the embryo has tightly adhered to epithelial cells, Eph–ephrin signals from the embryo can be continuously transduced to the endometrium [89]. The Eph–ephrin system was reported to induce reduction of the tight junction of epithelial cells via claudin 4, 5, and zonula occludens-1 [90,91]. The Eph–ephrin system was also found to promote cell attachment to the extracellular matrix [92]. Thus, we considered that this system reduces the endometrial epithelial barrier without destroying the connection between the embryo-attached epithelial cells and basement membrane [89] (Figure 3). On immunohistochemical study, Eph A1 and 4 were expressed on the luminal and glandular epithelium. Eph A2 was expressed on the pinopode-like sites of luminal epithelial cells [89]. Ephrin A1 induces phosphorylation in EphA2 and A4 in Ishikawa cells, a human endometrial carcinoma-derived cell line. Biological assays also revealed that the Eph A–ephrin A interaction induces cell attachment and intercellular dissociation in Ishikawa cells [93]. Ephrin A1-induced cell attachment was associated with the phosphorylation of focal adhesion kinase even in the presence of EDTA, suggesting the involvement of certain Ca ion-independent molecules. In contrast, EphA1-stimulation did not induce attachment of Ishikawa cells.

In the porcine uterus, the expression of Eph A1, A2, A4, and A7 was strongly detected in endometrial epithelial cells during early pregnancy. Ephrin A1 stimulated the proliferation of endometrial luminal epithelial cells via the activation of phosphoinositide 3-kinase (PI3K) and mitogen-activated protein kinase (MAPK) signaling proteins, suggesting that ephrin A1 is involved in the interactions between porcine blastocysts and endometrial luminal epithelial cells by activating PI3K and MAPK signal transduction pathways [94].

Notably, we observed that both EPH A1 and ephrin A4 signals promoted the invasion of a human choriocarcinoma-derived cell line, JEG-3 cells, without affecting cell proliferation [95]. It was also proposed that Eph A2 promotes the invasion and proliferation of the human extravillous trophoblast (EVT) through the ephrin-A1 ligand [96].

Based on these findings, we propose that the ephrin A signal induces intercellular dissociation and adhesion to the basement membrane in endometrial epithelial cells as embryo signals and suggest that this signal facilitates subsequent trophoblast invasion (Figure 3).

## 3. Positive Role of the Immune System in Embryo Implantation and Placentation

### 3.1. Regulation of Endometrial Receptivity and Embryo Invasion by Embryonic Signals

It is widely accepted that cytokine networks function in the establishment of endometrial receptivity, suggesting that the immune system plays an important role in embryo–maternal cross-talk [97,98,99,100]. In mice, seminal fluid was demonstrated to affect immune cells to induce endometrial differentiation and promote embryo implantation by activating inflammation and inducing immunological changes in the female reproductive tract, which facilitate endometrial receptivity [101,102]. The significance of this mechanism in women is an important subject to improve the outcome of in vitro fertilization and embryo transfer (IVF-ET) therapy [103].

On the other hand, we found that the intravenous administration of splenocytes from early pregnancy induced successful implantation in pseudo-pregnant day 2 recipient mice by promoting endometrial differentiation, which is suitable for embryo implantation [104]. In the delayed implantation model, the administration of splenocytes induced LIF expression in the uterus and subsequent embryo implantation [105]. In contrast to pregnant mice, splenocytes from pseudo-pregnant mice had no significant effects. As pseudo-pregnant recipient mice had been sensitized with the seminal fluid of vasectomized male mice [106], we considered that developing embryos in the Fallopian tube and uterus affect the maternal immune function to prepare suitable uterine conditions for implantation [107] and proposed the presence of the dual control of endometrial differentiation before embryo attachment via the endocrine and immune systems by embryonic signals [108,109,110].

The next question is how the maternal immune system recognizes the presence of a developing embryo before implantation and distinguishes the developing embryo from nonfertilized eggs and/or other organisms in the female genital tract [107]. To achieve this, the developing embryo should convey species- and embryo-specific signals, i.e., embryonic signals, to the maternal immune system. Early pregnancy factors that inhibit T-cell-induced rosette formation were reported to be detected just after fertilization [111]. Later, several substances, such as platelet activating factor, thioredoxin, and chaperonin 10, were proposed as early pregnancy factors [112,113,114]. It was also reported that the embryo-derived pre-implantation factor (PIF), a novel embryo-specific 18-kDa peptide that is specifically expressed on the fetus and placenta [115], was secreted at the two-cell stage and was able to be detected in the maternal sera prior to implantation [116]. Its many biological roles are under investigation [115,117,118].

Importantly, early pregnancy factor (EPF) is not species- or embryo-specific, whereas PIF is not species-specific to the maternal immune system. Consequently, as a new candidate for the embryonic signal before implantation, we paid attention to the zona pellucida (ZP) because it functions in the species- and oocyte-specific binding of spermatozoa [119]. As developing embryos actively degrade ZP, which is an abundant store of species- and oocyte-specific glycoproteins, from fertilization to hatching, we proposed that degradation products of ZP, including their sugar moieties, are utilized as intrinsic embryonic signals, which transmit information about the presence of the developing embryo to the maternal immune system in the reproductive tract [107].

As described above, hCG, a species- and embryo-specific glycoprotein, was proposed to directly affect the endometrial function as an embryonic signal after hatching [17,18,19,20,21,23]. Notably, this leading embryonic signal is also considered to induce an endometrial immune environment that accepts allograft implantation of the embryo, inducing fetal immune tolerance [120,121,122]. Approximately half a century ago, urinary hCG was demonstrated to suppress immune reactions, leading to the proposal of an essential role of hCG in the induction of immune tolerance to the fetus [123]. However, the following studies demonstrated that highly purified hCG did not have immunosuppressive effects [124]. Since then, the effects of hCG on immune cells remained controversial for a long time. Based on this background, we reported that recombinant hCG binds and activates CD14-positive monocytes to promote IL-8 production partially through the nuclear factor-kappa B (NF-κB) pathway at relatively high concentrations of more than 10 IU/mL. This hCG-induced IL-8 production was inhibited by the exogenous excess of D-mannose, suggesting that hCG regulates the peripheral blood mononuclear cell (PBMC) function through sugar chain receptors [125]. Later, it was demonstrated that a high concentration of hCG regulates uterine NK cell proliferation via mannose receptors rather than by luteinizing hormone (LH)/hCG receptors [126]. The carbohydrate chains of urinary hCG are largely cleaved before urine production [127]. Accordingly, the possible involvement of a carbohydrate-mediated primitive mechanism in the maternal response by the immune system may explain the previous discrepancy in the immunosuppressive effects on immune cells between urinary crude hCG and highly purified hCG and the reason why such a high concentration of hCG is necessary to maintain normal pregnancy [125].

In contrast to the above lectin–glycan interaction, hCG induces regulatory T-cells to migrate to the trophoblast through LH/hCG receptors that are expressed on regulatory T-cells [120]. Subsequent studies suggested the essential role of hCG in pregnancy-induced immune tolerance and embryo implantation, regulating the establishment of an adequate embryo–endometrial relationship [121,128]. In addition, CD19+CD24(high+)CD27+ regulatory B-cells were demonstrated to produce IL-10 by hCG stimulation via LH/hCG receptors [129]. This type of regulatory B-cell was proposed to mediate the positive effects of hCG on the immune environment during pregnancy [130,131].

In invasion assays using the murine embryo and BeWo cells, peripheral blood mononuclear cells (PBMCs) derived from women in early pregnancy promoted the murine trophectoderm and BeWo cell invasion more than those obtained from nonpregnant women. Importantly, when PBMCs from nonpregnant women were incubated with hCG, hCG-treated PBMCs promoted invasion more than nontreated PBMCs by soluble chemoattractive factors derived from PBMCs, suggesting that hCG alters PBMC functions to facilitate embryo implantation [132,133]. Later, similar effects of hCG on PBMCs to promote trophoblast invasion were reported using JAR cells, a cell line established from a human choriocarcinoma, inducing increases in matrix metalloproteinase (MMP)-2, MMP-9, and VEGF, and decreases in tissue inhibitor of metalloproteinase (TIMP)-1 and TIMP-2 expression [134].

Based on these findings, we propose that hCG stimulates maternal immune cells at the implantation site through lectin–glycan interaction, which in turn, promotes embryo attachment and invasion based on cooperation between the endocrine and immune systems [110].

### 3.2. Regulation of Extravillous Trophoblast (EVT) Invasion by Embryonic Signals

During human placental formation, the cytotrophoblast differentiates into EVT in the anchoring villi and invades the endometrial stromal tissues as a single cell, reconstructing maternal spiral arteries. The reduction of arterial contractility caused by arterial reconstruction facilitates adequate maternal blood flow into the intervillous spaces [135]. Failure of this process will lead to an insufficient blood supply and cause placental dysfunction and preeclampsia in the late stage of pregnancy [136].

As a representative embryonic signal during human trophoblast invasion, hCG was initially reported to directly reduce trophoblast invasion by inhibiting the enzyme activity of urokinase-plasminogen activator [137]. However, subsequent studies demonstrated that hCG increased the invasion and migration of JEG-3 cells, a trophoblastic cell line derived from choriocarcinoma [138]. hCG was also reported to increase cell migration of an EVT cell line, HTR-8 SVneo cells, through an insulin-like growth factor-II axis [139]. Later, hyperglycosylated hCG secreted by the invasive EVT, but not hCG produced by the syncytiotrophoblast, was found to promote trophoblast invasion [140]. It was also reported that hCG, hCGβ, and their hyperglycosylated forms stimulate the invasion of JEG-3 cells through an independent pathway involving the classical LH/hCG-receptor [141]. As described above, hCG-stimulated immune cells were demonstrated to promote trophoblast invasion by secreting chemoattractants through a paracrine mechanism [132,133,134]. Of note, human endometrial stromal cells were reported to increase the invasion of HTR-8/SVneo cells under hCG stimulation, suggesting paracrine effects of hCG on trophoblast invasion through endometrial stromal cells and local immune cells [115].

As a novel embryonic signal candidate, we identified a new cell surface aminopeptidase, initially named ‘laeverin’, which belongs to the M1 peptidase family [142], and was later termed ‘aminopeptidase Q’ [143]. Members of the M1 family of aminopeptidases share a common peptide-binding site (GXMEN) and peptidase activity motif (HEXXHX18E). Primate laeverin has a unique peptide-binding motif (HXMEN) where the first glycine (Gly) residue is substituted with histidine (His) [143], inducing significant changes in substrate specificity toward natural peptide hormones [144]. Laeverin is specifically expressed on EVT in the placenta from early and term pregnancy. In primary villous explant cultures, laeverin expression was induced on the cell surface of the outgrowing EVT, and secretion of soluble laeverin was detected in the culture media. The invasion of EVT isolated from primary culture was suppressed by the reduction of laeverin mRNA expression, whereas the soluble form of recombinant laeverin promoted EVT invasion, suggesting a regulatory role of laeverin in EVT invasion [145]. Similar to HLA-G, the expression of laeverin is specifically limited to EVT [142]. The co-expression of HLA-G and laeverin suggests specific roles of laeverin in the regulation of immune tolerance, which should be clarified in the future.

## 4. Topics of Clinical Application of Immune Therapy

At present, implantation failure is one of the most important clinical problems in IVF treatment. It should be noted that IVF therapy skips a large part of the maternal immune recognition process when the developing embryo is present in the female genital tract. Consequently, to achieve successful implantation, the precise assessment and accurate control of an active state of maternal immune tolerance mediated by regulatory T-cells is considered to be necessary [146]. Recently, personalization of immune treatment was recommended based on uterine immune profiles such as low immune activation or immune overactivation [147]. Currently, use of the new immunosuppressive agents tacrolimus and cyclosporine, which minimize immune rejection of transplanted organs, has been proposed as useful for infertile patients with immune overactivation [148,149]. When infertile patients with overactive immune conditions were carefully selected, tacrolimus was reported to improve the pregnancy outcome [149,150,151]. To support this approach, a common mTOR (mammalian target of rapamycin) inhibitor, sirolimus, which effectively prevents allograft rejection, was recently demonstrated to improve clinical pregnancy and live birth rates after repeated IVF therapy failures by reducing the Th17/Treg cell ratio [152].

In contrast to immunosuppressive therapy, we previously developed a novel therapy using autologous PBMCs. Briefly, PBMCs are isolated from patients and incubated with hCG, an embryonic signal, in order to sensitize them. The activated PBMCs are administered into the uterine cavity to induce adequate endometrial differentiation three days before blastocyst transfer. This treatment effectively improved the pregnancy and implantation rates in patients with four or more repeated IVF therapy failures [153]. The intrauterine administration of corticotropin-releasing hormone (CRH)-treated autologous PBMCs was also demonstrated to improve clinical pregnancy rates of patients with repeated implantation failure [154]. Currently, the effectiveness of PBMC therapy has been positively reported [155,156,157,158].

**Figure 4:**
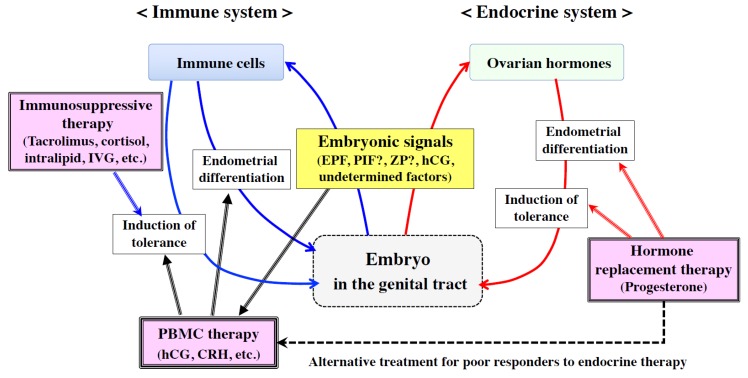
Strategy of immune therapy using embryonic signals. Embryonic signals from the genital tract act on both endocrine (red arrows) and immune (blue arrows) systems to induce endometrial differentiation and immune tolerance. To assist the endocrine system, hormone replacement therapy was recommended to promote endometrial receptivity (red triple lines), whereas to minimize immune rejection, immunosuppressive agents were administered to elicit immune tolerance (blue triple line). On the other hand, to induce sufficient endometrial differentiation and receptivity in infertile patients who poorly respond to endocrine stimulation (black dotted line), intrauterine administration of embryonic signal-activated peripheral blood mononuclear cells (PBMCs) is one of the promising approaches that may induce favorable endometrial differentiation and inflammatory reactions together with a favorable immune environment for embryo implantation (black triple lines).

Embryonic signals elicit two different effects: induction of immune tolerance and adequate local inflammation; the former protects against immune rejection, whereas the latter contributes to endometrial differentiation and maternal tissue remodeling during embryo implantation and placentation [108,109,110] (Figure 4). At present, there are few effective therapies to induce adequate endometrial differentiation and receptivity for infertile patients who poorly respond to endocrine stimulation. In this regard, the intrauterine administration of embryonic signal-activated PBMCs is one of the promising approaches to induce favorable endometrial differentiation and inflammatory reactions together with a favorable immune environment for embryo implantation [108] (Figure 4). It should also be noted that PBMCs can change the function or structure of endometrial surface molecules by secreting proteases. Since PBMCs have been suggested to move from the uterine cavity toward endometrial stromal tissue, they may create several pathways for subsequent embryo attachment and invasion.

## 5. Conclusions

In conclusion, we propose the involvement of repulsive molecules, the Eph–ephrin system, in endometrial receptivity for embryo implantation, together with adhesion-promoting and adhesion-inhibiting molecules. This Eph–ephrin system may contribute to opening tight junctions among endometrial epithelial cells to induce embryo invasion toward endometrial stroma tissues, suggesting that Eph–ephrin molecules are new candidates for embryonic signals that induce suitable local inflammation for embryo invasion. Embryonic signals also elicit two different effects from the immune system: induction of immune tolerance that protects against immune rejection, and sufficient local inflammation that functions in endometrial differentiation and remodeling during embryo implantation and placentation. Further clarification of the precise roles and possible clinical usage of embryonic signal candidates, such as PIF, ZP degradation products, or laeverin, will help improve immunotherapy to minimize implantation failure in the future.

## Abbreviations

ALCAM	activated leukocyte cell adhesion molecule
BM	basement membrane
ECM	extracellular matrix
EMT	epithelial–mesenchymal transition
EVT	extravillous trophoblast
hCG	human chorionic gonadotropin
IGFBP-1	insulin-like growth factor binding protein-1
IL	interleukin
IVF-ET	in vitro fertilization and embryo transfer
LIF	leukemia inhibitory factor
M-CSF	macrophage colony stimulating factor
MMP	matrix metalloproteinase
mTOR	mammalian target of rapamycin
PBMC	peripheral blood mononuclear cell
PIF	pre-implantation factor
PTENP1	phosphatase and tensin homolog pseudogene 1
TIMP	tissue inhibitor of metalloproteinase
VCAM1	vascular cell adhesion molecule 1
VEGF	vascular endothelial growth factor
ZP	zona pellucida

## References

[1] Wetendorf M., DeMayo F.J. (2012). The progesterone receptor regulates implantation, decidualization, and glandular development via a complex paracrine signaling network. Mol. Cell Endocrinol..

[2] Large M.J., DeMayo F.J. (2012). The regulation of embryo implantation and endometrial decidualization by progesterone receptor signaling. Mol. Cell Endocrinol..

[3] Yoshinaga K. (1988). Uterine receptivity for blastocyst implantation. Ann. N. Y. Acad. Sci..

[4] Sarantis L., Roche D., Psychoyos A. (1988). Displacement of receptivity for nidation in the rat by the progesterone antagonist RU 486: A scanning electron microscopy study. Hum. Reprod..

[5] Psychoyos A. (1993). The Implantation Window: BASIC and Clinical Aspects.

[6] Fukui Y., Hirota Y., Matsuo M., Gebril M., Akaeda S., Hiraoka T., Osuga Y. (2019). Uterine receptivity, embryo attachment, and embryo invasion: Multistep processes in embryo implantation. Reprod. Med. Biol..

[7] Edwards R.G. (1988). Human uterine endocrinology and the implantation window. Ann. N. Y. Acad. Sci..

[8] Lessey B.A. (2000). The role of the endometrium during embryo implantation. Hum. Reprod..

[9] Kosaka K., Fujiwara H., Tatsumi K., Yoshioka S., Higuchi T., Sato Y., Nakayama T., Fujii S. (2003). Human peripheral blood mononuclear cells enhance cell-cell interaction between human endometrial epithelial cells and BeWo-cell spheroids. Hum. Reprod..

[10] Tur-Kaspa I., Confino E., Dudkiewicz A.B., Myers S.A., Friberg J., Gleicher N. (1990). Ovarian stimulation protocol for in vitro fertilization with gonadotropin-releasing hormone agonist widens the implantation window. Fertil. Steril..

[11] Murphy C.R. (2000). Understanding the apical surface markers of uterine receptivity: Pinopods-or uterodomes?. Hum. Reprod..

[12] Quinn K.E., Matson B.C., Wetendorf M., Caron K.M. (2019). Pinopodes: Recent advancements, current perspectives, and future directions. Mol. Cell Endocrinol..

[13] Tabibzadeh S., Babaknia A. (1995). The signals and molecular pathways involved in implantation, a symbiotic interaction between blastocyst and endometrium involving adhesion and tissue invasion. Hum. Reprod..

[14] Simon C., Dominguez F., Remohi J., Pellicer A. (2001). Embryo effects in human implantation: Embryonic regulation of endometrial molecules in human implantation. Ann. N. Y. Acad. Sci..

[15] Daikoku T., Cha J., Sun X., Tranguch S., Xie H., Fujita T., Hirota Y., Lydon J., DeMayo F., Maxson R. (2011). Conditional deletion of Msx homeobox genes in the uterus inhibits blastocyst implantation by altering uterine receptivity. Dev. Cell.

[16] Namiki T., Ito J., Kashiwazaki N. (2018). Molecular mechanisms of embryonic implantation in mammals: Lessons from the gene manipulation of mice. Reprod. Med. Biol..

[17] Fazleabas A.T., Kim J.J., Strakova Z. (2004). Implantation: Embryonic signals and the modulation of the uterine environment—A review. Placenta.

[18] Rao C.V., Lei Z.M. (2007). The past, present and future of nongonadal LH/hCG actions in reproductive biology and medicine. Mol. Cell Endocrinol..

[19] Fazleabas A.T., Donnelly K.M., Srinivasan S., Fortman J.D., Miller J.B. (1999). Modulation of the baboon (Papio anubis) uterine endometrium by chorionic gonadotrophin during the period of uterine receptivity. Proc. Natl. Acad. Sci. USA.

[20] Licht P., Russu V., Wildt L. (2001). On the role of human chorionic gonadotropin (hCG) in the embryo-endometrial microenvironment: Implications for differentiation and implantation. Semin. Reprod. Med..

[21] Licht P., Fluhr H., Neuwinger J., Wallwiener D., Wildt L. (2007). Is human chorionic gonadotropin directly involved in the regulation of human implantation?. Mol. Cell Endocrinol..

[22] Evans J. (2016). Hyperglycosylated hCG: A Unique Human Implantation and Invasion Factor. Am. J. Reprod. Immunol..

[23] Makrigiannakis A., Vrekoussis T., Zoumakis E., Kalantaridou S.N., Jeschke U. (2017). The Role of HCG in Implantation: A Mini-Review of Molecular and Clinical Evidence. Int. J. Mol. Sci..

[24] Cuman C., Van Sinderen M., Gantier M.P., Rainczuk K., Sorby K., Rombauts L., Osianlis T., Dimitriadis E. (2015). Human Blastocyst Secreted microRNA Regulate Endometrial Epithelial Cell Adhesion. EBioMedicine.

[25] Es-Haghi M., Godakumara K., Haling A., Lattekivi F., Lavrits A., Viil J., Andronowska A., Nafee T., James V., Jaakma U. (2019). Specific trophoblast transcripts transferred by extracellular vesicles affect gene expression in endometrial epithelial cells and may have a role in embryo-maternal crosstalk. Cell Commun. Signal..

[26] Takamura M., Zhou W., Rombauts L., Dimitriadis E. (2020). The long noncoding RNA PTENP1 regulates human endometrial epithelial adhesive capacity in vitro: Implications in infertility. Biol. Reprod..

[27] Sugihara K., Sugiyama D., Byrne J., Wolf D.P., Lowitz K.P., Kobayashi Y., Kabir-Salmani M., Nadano D., Aoki D., Nozawa S. (2007). Trophoblast cell activation by trophinin ligation is implicated in human embryo implantation. Proc. Natl. Acad. Sci. USA.

[28] Fukuda M.N., Sugihara K. (2007). Signal transduction in human embryo implantation. Cell Cycle.

[29] Fukuda M.N., Sugihara K. (2008). An integrated view of L-selectin and trophinin function in human embryo implantation. J. Obstet. Gynaecol. Res..

[30] Genbacev O.D., Prakobphol A., Foulk R.A., Krtolica A.R., Ilic D., Singer M.S., Yang Z.Q., Kiessling L.L., Rosen S.D., Fisher S.J. (2003). Trophoblast L-selectin-mediated adhesion at the maternal-fetal interface. Science.

[31] Lai T.H., Zhao Y., Shih Ie M., Ho C.L., Bankowski B., Vlahos N. (2006). Expression of L-selectin ligands in human endometrium during the implantation window after controlled ovarian stimulation for oocyte donation. Fertil. Steril..

[32] Foulk R.A., Zdravkovic T., Genbacev O., Prakobphol A. (2007). Expression of L-selectin ligand MECA-79 as a predictive marker of human uterine receptivity. J. Assist. Reprod. Genet..

[33] Feng Y., Ma X., Deng L., Yao B., Xiong Y., Wu Y., Wang L., Ma Q., Ma F. (2017). Role of selectins and their ligands in human implantation stage. Glycobiology.

[34] Lessey B.A., Castelbaum A.J., Sawin S.W., Sun J. (1995). Integrins as markers of uterine receptivity in women with primary unexplained infertility. Fertil. Steril..

[35] Lessey B.A., Arnold J.T. (1998). Paracrine signaling in the endometrium: Integrins and the establishment of uterine receptivity. J. Reprod. Immunol..

[36] Chung T.W., Park M.J., Kim H.S., Choi H.J., Ha K.T. (2016). Integrin alphaVbeta3 and alphaVbeta5 are required for leukemia inhibitory factor-mediated the adhesion of trophoblast cells to the endometrial cells. Biochem. Biophys. Res. Commun..

[37] Elnaggar A., Farag A.H., Gaber M.E., Hafeez M.A., Ali M.S., Atef A.M. (2017). AlphaVBeta3 Integrin expression within uterine endometrium in unexplained infertility: A prospective cohort study. BMC Women’s Health.

[38] Aplin J.D., Seif M.W., Graham R.A., Hey N.A., Behzad F., Campbell S. (1994). The endometrial cell surface and implantation. Expression of the polymorphic mucin MUC-1 and adhesion molecules during the endometrial cycle. Ann. N. Y. Acad. Sci..

[39] Albers A., Thie M., Hohn H.P., Denker H.W. (1995). Differential expression and localization of integrins and CD44 in the membrane domains of human uterine epithelial cells during the menstrual cycle. Acta Anat. (Basel).

[40] Berneau S.C., Ruane P.T., Brison D.R., Kimber S.J., Westwood M., Aplin J.D. (2019). Investigating the role of CD44 and hyaluronate in embryo-epithelial interaction using an in vitro model. Mol. Hum. Reprod..

[41] Simon C., Gimeno M.J., Mercader A., O’Connor J.E., Remohi J., Polan M.L., Pellicer A. (1997). Embryonic regulation of integrins beta 3, alpha 4, and alpha 1 in human endometrial epithelial cells in vitro. J. Clin. Endocrinol. Metab..

[42] Takahashi H., Takizawa T., Matsubara S., Ohkuchi A., Kuwata T., Usui R., Matsumoto H., Sato Y., Fujiwara H., Okamoto A. (2014). Extravillous trophoblast cell invasion is promoted by the CD44-hyaluronic acid interaction. Placenta.

[43] Miyazaki Y., Horie A., Tani H., Ueda M., Okunomiya A., Suginami K., Kondoh E., Baba T., Konishi I., Shinomura T. (2019). Versican V1 in human endometrial epithelial cells promotes BeWo spheroid adhesion in vitro. Reproduction.

[44] Bai R., Bai H., Kuse M., Ideta A., Aoyagi Y., Fujiwara H., Okuda K., Imakawa K., Sakurai T. (2014). Involvement of VCAM1 in the bovine conceptus adhesion to the uterine endometrium. Reproduction.

[45] Park K.R., Inoue T., Ueda M., Hirano T., Higuchi T., Maeda M., Konishi I., Fujiwara H., Fujii S. (2000). CD9 is expressed on human endometrial epithelial cells in association with integrins alpha(6), alpha(3) and beta(1). Mol. Hum. Reprod..

[46] Liu W.M., Cao Y.J., Yang Y.J., Li J., Hu Z., Duan E.K. (2006). Tetraspanin CD9 regulates invasion during mouse embryo implantation. J. Mol. Endocrinol..

[47] Dominguez F., Simon C., Quinonero A., Ramirez M.A., Gonzalez-Munoz E., Burghardt H., Cervero A., Martinez S., Pellicer A., Palacin M. (2010). Human endometrial CD98 is essential for blastocyst adhesion. PLoS ONE.

[48] Iwai M., Hamatani T., Nakamura A., Kawano N., Kanai S., Kang W., Yoshii N., Odawara Y., Yamada M., Miyamoto Y. (2019). Membrane protein CD9 is repositioned and released to enhance uterine function. Lab. Investig..

[49] Hirano T., Higuchi T., Katsuragawa H., Inoue T., Kataoka N., Park K.R., Ueda M., Maeda M., Fujiwara H., Fujii S. (1999). CD9 is involved in invasion of human trophoblast-like choriocarcinoma cell line, BeWo cells. Mol. Hum. Reprod..

[50] Hirano T., Higuchi T., Ueda M., Inoue T., Kataoka N., Maeda M., Fujiwara H., Fujii S. (1999). CD9 is expressed in extravillous trophoblasts in association with integrin alpha3 and integrin alpha5. Mol. Hum. Reprod..

[51] Matsumoto H., Sato Y., Horie A., Suginami K., Tani H., Hattori A., Araki Y., Kagami K., Konishi I., Fujiwara H. (2016). CD9 suppresses human extravillous trophoblast invasion. Placenta.

[52] Hey N.A., Graham R.A., Seif M.W., Aplin J.D. (1994). The polymorphic epithelial mucin MUC1 in human endometrium is regulated with maximal expression in the implantation phase. J. Clin. Endocrinol. Metab..

[53] Aplin J.D., Hey N.A., Graham R.A. (1998). Human endometrial MUC1 carries keratan sulfate: Characteristic glycoforms in the luminal epithelium at receptivity. Glycobiology.

[54] Meseguer M., Aplin J.D., Caballero-Campo P., O’Connor J.E., Martin J.C., Remohi J., Pellicer A., Simon C. (2001). Human endometrial mucin MUC1 is up-regulated by progesterone and down-regulated in vitro by the human blastocyst. Biol. Reprod..

[55] Gipson I.K., Blalock T., Tisdale A., Spurr-Michaud S., Allcorn S., Stavreus-Evers A., Gemzell K. (2008). MUC16 is lost from the uterodome (pinopode) surface of the receptive human endometrium: In vitro evidence that MUC16 is a barrier to trophoblast adherence. Biol. Reprod..

[56] Paria B.C., Reese J., Das S.K., Dey S.K. (2002). Deciphering the cross-talk of implantation: Advances and challenges. Science.

[57] Chen Q., Zhang Y., Elad D., Jaffa A.J., Cao Y., Ye X., Duan E. (2013). Navigating the site for embryo implantation: Biomechanical and molecular regulation of intrauterine embryo distribution. Mol. Aspects Med..

[58] Imakawa K., Bai R., Kusama K. (2018). Integration of molecules to construct the processes of conceptus implantation to the maternal endometrium. J. Anim. Sci..

[59] Sanchez J.M., Mathew D.J., Passaro C., Fair T., Lonergan P. (2018). Embryonic maternal interaction in cattle and its relationship with fertility. Reprod. Domest. Anim..

[60] Simintiras C.A., Sanchez J.M., McDonald M., Lonergan P. (2019). The biochemistry surrounding bovine conceptus elongationdagger. Biol. Reprod..

[61] Yoshinaga K., Adams C.E. (1966). Delayed implantation in the spayed, progesterone treated adult mouse. J. Reprod. Fertil..

[62] Hamatani T., Daikoku T., Wang H., Matsumoto H., Carter M.G., Ko M.S., Dey S.K. (2004). Global gene expression analysis identifies molecular pathways distinguishing blastocyst dormancy and activation. Proc. Natl. Acad. Sci. USA.

[63] Fujiwara H., Yoshioka S., Tatsumi K., Kosaka K., Satoh Y., Nishioka Y., Egawa M., Higuchi T., Fujii S. (2002). Human endometrial epithelial cells express ephrin A1: Possible interaction between human blastocysts and endometrium via Eph-ephrin system. J. Clin. Endocrinol. Metab..

[64] Pasquale E.B. (2005). Eph receptor signalling casts a wide net on cell behaviour. Nat. Rev. Mol. Cell Biol..

[65] Kania A., Klein R. (2016). Mechanisms of ephrin-Eph signalling in development, physiology and disease. Nat. Rev. Mol. Cell Biol..

[66] Darling T.K., Lamb T.J. (2019). Emerging Roles for Eph Receptors and Ephrin Ligands in Immunity. Front. Immunol..

[67] Adams R.H., Klein R. (2000). Eph receptors and ephrin ligands. essential mediators of vascular development. Trends. Cardiovasc. Med..

[68] Wolf K., Hu H., Isaji T., Dardik A. (2019). Molecular identity of arteries, veins, and lymphatics. J. Vasc. Surg..

[69] Klein R. (2009). Bidirectional modulation of synaptic functions by Eph/ephrin signaling. Nat. Neurosci..

[70] Fujii H., Tatsumi K., Kosaka K., Yoshioka S., Fujiwara H., Fujii S. (2006). Eph-ephrin A system regulates murine blastocyst attachment and spreading. Dev. Dyn..

[71] Fu Y., Fu J., Ren Q., Chen X., Wang A. (2012). Expression of Eph A molecules during swine embryo implantation. Mol. Biol. Rep..

[72] Fu Y., Li L., Fang X., Li B., Zhao W., Zhou L., Ren S. (2018). Investigation of Eph-ephrin A1 in the regulation of embryo implantation in sows. Reprod. Domest. Anim..

[73] Fu Y., Knox R.V., Li L., Ren S. (2018). Differential gene expression of Eph-ephrin A1 and LEPR-LEP with high or low number of embryos in pigs during implantation. Reprod. Domest. Anim..

[74] Fujiwara H., Tatsumi K., Kosaka K., Sato Y., Higuchi T., Yoshioka S., Maeda M., Ueda M., Fujii S. (2003). Human blastocysts and endometrial epithelial cells express activated leukocyte cell adhesion molecule (ALCAM/CD166). J. Clin. Endocrinol. Metab..

[75] Bowen M.A., Aruffo A.A., Bajorath J. (2000). Cell surface receptors and their ligands: In vitro analysis of CD6-CD166 interactions. Proteins.

[76] Swart G.W. (2002). Activated leukocyte cell adhesion molecule (CD166/ALCAM): Developmental and mechanistic aspects of cell clustering and cell migration. Eur. J. Cell Biol..

[77] Gilsanz A., Sanchez-Martin L., Gutierrez-Lopez M.D., Ovalle S., Machado-Pineda Y., Reyes R., Swart G.W., Figdor C.G., Lafuente E.M., Cabanas C. (2013). ALCAM/CD166 adhesive function is regulated by the tetraspanin CD9. Cell Mol. Life Sci..

[78] Haouzi D., Dechaud H., Assou S., Monzo C., de Vos J., Hamamah S. (2011). Transcriptome analysis reveals dialogues between human trophectoderm and endometrial cells during the implantation period. Hum. Reprod..

[79] Zen K., Parkos C.A. (2003). Leukocyte-epithelial interactions. Curr. Opin. Cell Biol..

[80] Agace W.W., Higgins J.M., Sadasivan B., Brenner M.B., Parker C.M. (2000). T-lymphocyte-epithelial-cell interactions: Integrin alpha(E)(CD103)beta(7), LEEP-CAM and chemokines. Curr. Opin. Cell Biol..

[81] Luissint A.C., Parkos C.A., Nusrat A. (2016). Inflammation and the Intestinal Barrier: Leukocyte-Epithelial Cell Interactions, Cell Junction Remodeling, and Mucosal Repair. Gastroenterology.

[82] Matthews J.D., Weight C.M., Parkos C.A. (2014). Leukocyte-epithelial interactions and mucosal homeostasis. Toxicol. Pathol..

[83] Rescigno M., Urbano M., Valzasina B., Francolini M., Rotta G., Bonasio R., Granucci F., Kraehenbuhl J.P., Ricciardi-Castagnoli P. (2001). Dendritic cells express tight junction proteins and penetrate gut epithelial monolayers to sample bacteria. Nat. Immunol..

[84] Owusu-Akyaw A., Krishnamoorthy K., Goldsmith L.T., Morelli S.S. (2019). The role of mesenchymal-epithelial transition in endometrial function. Hum. Reprod. Update.

[85] Imakawa K., Bai R., Fujiwara H., Ideta A., Aoyagi Y., Kusama K. (2017). Continuous model of conceptus implantation to the maternal endometrium. J. Endocrinol..

[86] Imakawa K., Bai R., Fujiwara H., Kusama K. (2016). Conceptus implantation and placentation: Molecules related to epithelial-mesenchymal transition, lymphocyte homing, endogenous retroviruses, and exosomes. Reprod. Med. Biol..

[87] Uchida H., Maruyama T., Nishikawa-Uchida S., Oda H., Miyazaki K., Yamasaki A., Yoshimura Y. (2012). Studies using an in vitro model show evidence of involvement of epithelial-mesenchymal transition of human endometrial epithelial cells in human embryo implantation. J. Biol. Chem..

[88] Yoshinaga K. (2008). Review of factors essential for blastocyst implantation for their modulating effects on the maternal immune system. Semin. Cell Dev. Biol..

[89] Fujii H., Fujiwara H., Horie A., Sato Y., Konishi I. (2011). Ephrin A1 induces intercellular dissociation in Ishikawa cells: Possible implication of the Eph-ephrin A system in human embryo implantation. Hum. Reprod.

[90] Tanaka M., Kamata R., Sakai R. (2005). EphA2 phosphorylates the cytoplasmic tail of Claudin-4 and mediates paracellular permeability. J. Biol. Chem..

[91] Chen F., Liu Z., Peng W., Gao Z., Ouyang H., Yan T., Ding S., Cai Z., Zhao B., Mao L. (2018). Activation of EphA4 induced by EphrinA1 exacerbates disruption of the blood-brain barrier following cerebral ischemia-reperfusion via the Rho/ROCK signaling pathway. Exp. Ther. Med..

[92] Carter N., Nakamoto T., Hirai H., Hunter T. (2002). EphrinA1-induced cytoskeletal re-organization requires FAK and p130(cas). Nat. Cell Biol..

[93] Fujii H., Fujiwara H., Horie A., Suginami K., Sato Y., Konishi I. (2011). EphrinA1 stimulates cell attachment and inhibits cell aggregation through the EphA receptor pathway in human endometrial carcinoma-derived Ishikawa cells. Hum. Reprod..

[94] Lim W., Bae H., Bazer F.W., Song G. (2017). Functional Roles of Eph A-Ephrin A1 System in Endometrial Luminal Epithelial Cells During Early Pregnancy. J. Cell Physiol..

[95] Fujiwara H., Nishioka Y., Matsumoto H., Suginami K., Horie A., Tani H., Matsumura N., Baba T., Sato Y., Araki Y. (2013). Eph-ephrin A system regulates human choriocarcinoma-derived JEG-3 cell invasion. Int. J. Gynecol. Cancer.

[96] Yang Y., Min J. (2011). Effect of ephrin-A1/EphA2 on invasion of trophoblastic cells. J. Huazhong Univ. Sci. Technolog. Med. Sci..

[97] Simon C., Frances A., Piquette G., Hendrickson M., Milki A., Polan M.L. (1994). Interleukin-1 system in the materno-trophoblast unit in human implantation: Immunohistochemical evidence for autocrine/paracrine function. J. Clin. Endocrinol. Metab..

[98] Salamonsen L.A., Hannan N.J., Dimitriadis E. (2007). Cytokines and chemokines during human embryo implantation: Roles in implantation and early placentation. Semin. Reprod. Med..

[99] Saito S. (2000). Cytokine network at the feto-maternal interface. J. Reprod. Immunol..

[100] Robertson S.A., Chin P.Y., Glynn D.J., Thompson J.G. (2011). Peri-conceptual cytokines—Setting the trajectory for embryo implantation, pregnancy and beyond. Am. J. Reprod. Immunol..

[101] Schjenken J.E., Robertson S.A. (2014). Seminal fluid and immune adaptation for pregnancy—Comparative biology in mammalian species. Reprod. Domest. Anim..

[102] Robertson S.A. (2007). Seminal fluid signaling in the female reproductive tract: Lessons from rodents and pigs. J. Anim. Sci..

[103] Robertson S.A., Sharkey D.J. (2016). Seminal fluid and fertility in women. Fertil. Steril..

[104] Takabatake K., Fujiwara H., Goto Y., Nakayama T., Higuchi T., Maeda M., Mori T. (1997). Intravenous administration of splenocytes in early pregnancy changes the implantation window in mice. Hum. Reprod..

[105] Takabatake K., Fujiwara H., Goto Y., Nakayama T., Higuchi T., Fujita J., Maeda M., Mori T. (1997). Splenocytes in early pregnancy promote embryo implantation by regulating endometrial differentiation in mice. Hum. Reprod..

[106] Larson M.A. (2020). Embryo Transfer Surgery. Methods Mol. Biol..

[107] Fujiwara H., Araki Y., Toshimori K. (2009). Is the zona pellucida an intrinsic source of signals activating maternal recognition of the developing mammalian embryo?. J. Reprod. Immunol..

[108] Fujiwara H., Araki Y., Imakawa K., Saito S., Daikoku T., Shigeta M., Kanzaki H., Mori T. (2016). Dual Positive Regulation of Embryo Implantation by Endocrine and Immune Systems—Step-by-Step Maternal Recognition of the Developing Embryo. Am. J. Reprod. Immunol..

[109] Fujiwara H. (2009). Do circulating blood cells contribute to maternal tissue remodeling and embryo-maternal cross-talk around the implantation period?. Mol. Hum. Reprod..

[110] Fujiwara H. (2006). Immune cells contribute to systemic cross-talk between the embryo and mother during early pregnancy in cooperation with the endocrine system. Reprod. Med. Biol..

[111] Morton H., Hegh V., Clunie G.J. (1974). Immunosuppression detected in pregnant mice by rosette inhibition test. Nature.

[112] Clarke F.M. (1992). Identification of molecules and mechanisms involved in the ‘early pregnancy factor’ system. Reprod. Fertil. Dev..

[113] Morton H. (1998). Early pregnancy factor: An extracellular chaperonin 10 homologue. Immunol. Cell Biol..

[114] Chen Q., Zhu X., Chen R., Liu J., Liu P., Hu A., Wu L., Hua H., Yuan H. (2016). Early Pregnancy Factor Enhances the Generation and Function of CD4(+)CD25(+) Regulatory T Cells. Tohoku. J. Exp. Med..

[115] Stamatkin C.W., Roussev R.G., Stout M., Absalon-Medina V., Ramu S., Goodman C., Coulam C.B., Gilbert R.O., Godke R.A., Barnea E.R. (2011). PreImplantation Factor (PIF) correlates with early mammalian embryo development-bovine and murine models. Reprod. Biol. Endocrinol..

[116] Barnea E.R. (2007). Applying embryo-derived immune tolerance to the treatment of immune disorders. Ann. N. Y. Acad. Sci..

[117] Barnea E.R., Almogi-Hazan O., Or R., Mueller M., Ria F., Weiss L., Paidas M.J. (2015). Immune regulatory and neuroprotective properties of preimplantation factor: From newborn to adult. Pharmacol. Ther..

[118] Hayrabedyan S., Shainer R., Yekhtin Z., Weiss L., Almogi-Hazan O., Or R., Farnsworth C.L., Newsome S., Todorova K., Paidas M.J. (2019). Synthetic PreImplantation Factor (sPIF) induces posttranslational protein modification and reverses paralysis in EAE mice. Sci. Rep..

[119] Clark G.F. (2014). A role for carbohydrate recognition in mammalian sperm-egg binding. Biochem. Biophys. Res. Commun..

[120] Schumacher A., Brachwitz N., Sohr S., Engeland K., Langwisch S., Dolaptchieva M., Alexander T., Taran A., Malfertheiner S.F., Costa S.D. (2009). Human chorionic gonadotropin attracts regulatory T cells into the fetal-maternal interface during early human pregnancy. J. Immunol..

[121] Schumacher A., Heinze K., Witte J., Poloski E., Linzke N., Woidacki K., Zenclussen A.C. (2013). Human chorionic gonadotropin as a central regulator of pregnancy immune tolerance. J. Immunol..

[122] Schumacher A. (2017). Human Chorionic Gonadotropin as a Pivotal Endocrine Immune Regulator Initiating and Preserving Fetal Tolerance. Int. J. Mol. Sci..

[123] Adcock E.W., Teasdale T., August C.S., Cox S., Meschia G., Ballaglia T.C., Naughton M.A. (1973). Human chorionic gonadotropin: Its possible role in maternal lymphocyte suppression. Science.

[124] Muchmore A.V., Blaese R.M. (1977). Immunoregulatory properties of fractions from human pregnancy urine: Evidence that human chorionic gonadotropin is not responsible. J. Immunol..

[125] Kosaka K., Fujiwara H., Tatsumi K., Yoshioka S., Sato Y., Egawa H., Higuchi T., Nakayama T., Ueda M., Maeda M. (2002). Human chorionic gonadotropin (HCG) activates monocytes to produce interleukin-8 via a different pathway from luteinizing hormone/HCG receptor system. J. Clin. Endocrinol. Metab..

[126] Kane N., Kelly R., Saunders P.T., Critchley H.O. (2009). Proliferation of uterine natural killer cells is induced by human chorionic gonadotropin and mediated via the mannose receptor. Endocrinology.

[127] Cole L.A. (2007). Hyperglycosylated hCG. Placenta.

[128] Schumacher A., Zenclussen A.C. (2019). Human Chorionic Gonadotropin-Mediated Immune Responses That Facilitate Embryo Implantation and Placentation. Front. Immunol..

[129] Rolle L., Memarzadeh Tehran M., Morell-Garcia A., Raeva Y., Schumacher A., Hartig R., Costa S.D., Jensen F., Zenclussen A.C. (2013). Cutting edge: IL-10-producing regulatory B cells in early human pregnancy. Am. J. Reprod. Immunol..

[130] Guzman-Genuino R.M., Eldi P., Garcia-Valtanen P., Hayball J.D., Diener K.R. (2019). Uterine B Cells Exhibit Regulatory Properties During the Peri-Implantation Stage of Murine Pregnancy. Front. Immunol..

[131] Koushaeian L., Ghorbani F., Ahmadi M., Eghbal-Fard S., Zamani M., Danaii S., Yousefi B., Jadidi-Niaragh F., Hamdi K., Yousefi M. (2019). The role of IL-10-producing B cells in repeated implantation failure patients with cellular immune abnormalities. Immunol. Lett..

[132] Egawa H., Fujiwara H., Hirano T., Nakayama T., Higuchi T., Tatsumi K., Mori T., Fujii S. (2002). Peripheral blood mononuclear cells in early pregnancy promote invasion of human choriocarcinoma cell line, BeWo cells. Hum. Reprod..

[133] Nakayama T., Fujiwara H., Maeda M., Inoue T., Yoshioka S., Mori T., Fujii S. (2002). Human peripheral blood mononuclear cells (PBMC) in early pregnancy promote embryo invasion in vitro: HCG enhances the effects of PBMC. Hum. Reprod..

[134] Yu N., Yan W., Yin T., Wang Y., Guo Y., Zhou D., Xu M., Ding J., Yang J. (2015). HCG-Activated Human Peripheral Blood Mononuclear Cells (PBMC) Promote Trophoblast Cell Invasion. PLoS ONE.

[135] O’Tierney-Ginn P.F., Lash G.E. (2014). Beyond pregnancy: Modulation of trophoblast invasion and its consequences for fetal growth and long-term children’s health. J. Reprod. Immunol..

[136] Sato Y. (2019). Endovascular trophoblast and spiral artery remodeling. Mol. Cell Endocrinol..

[137] Yagel S., Geva T.E., Solomon H., Shimonovitz S., Reich R., Finci-Yeheskel Z., Mayer M., Milwidsky A. (1993). High levels of human chorionic gonadotropin retard first trimester trophoblast invasion in vitro by decreasing urokinase plasminogen activator and collagenase activities. J. Clin. Endocrinol. Metab..

[138] Zygmunt M., Hahn D., Munstedt K., Bischof P., Lang U. (1998). Invasion of cytotrophoblastic JEG-3 cells is stimulated by hCG in vitro. Placenta.

[139] Zygmunt M., McKinnon T., Herr F., Lala P.K., Han V.K. (2005). HCG increases trophoblast migration in vitro via the insulin-like growth factor-II/mannose-6 phosphate receptor. Mol. Hum. Reprod..

[140] Handschuh K., Guibourdenche J., Tsatsaris V., Guesnon M., Laurendeau I., Evain-Brion D., Fournier T. (2007). Human chorionic gonadotropin produced by the invasive trophoblast but not the villous trophoblast promotes cell invasion and is down-regulated by peroxisome proliferator-activated receptor-gamma. Endocrinology.

[141] Lee C.L., Chiu P.C., Hautala L., Salo T., Yeung W.S., Stenman U.H., Koistinen H. (2013). Human chorionic gonadotropin and its free beta-subunit stimulate trophoblast invasion independent of LH/hCG receptor. Mol. Cell Endocrinol..

[142] Fujiwara H., Higuchi T., Yamada S., Hirano T., Sato Y., Nishioka Y., Yoshioka S., Tatsumi K., Ueda M., Maeda M. (2004). Human extravillous trophoblasts express laeverin, a novel protein that belongs to membrane-bound gluzincin metallopeptidases. Biochem. Biophys. Res. Commun..

[143] Maruyama M., Hattori A., Goto Y., Ueda M., Maeda M., Fujiwara H., Tsujimoto M. (2007). Laeverin/aminopeptidase Q, a novel bestatin-sensitive leucine aminopeptidase belonging to the M1 family of aminopeptidases. J. Biol. Chem..

[144] Maruyama M., Arisaka N., Goto Y., Ohsawa Y., Inoue H., Fujiwara H., Hattori A., Tsujimoto M. (2009). Histidine 379 of human laeverin/aminopeptidase Q, a nonconserved residue within the exopeptidase motif, defines its distinctive enzymatic properties. J. Biol. Chem..

[145] Horie A., Fujiwara H., Sato Y., Suginami K., Matsumoto H., Maruyama M., Konishi I., Hattori A. (2012). Laeverin/aminopeptidase Q induces trophoblast invasion during human early placentation. Hum. Reprod..

[146] Robertson S.A., Care A.S., Moldenhauer L.M. (2018). Regulatory T cells in embryo implantation and the immune response to pregnancy. J. Clin. Investig..

[147] Ledee N., Petitbarat M., Chevrier L., Vitoux D., Vezmar K., Rahmati M., Dubanchet S., Gahery H., Bensussan A., Chaouat G. (2016). The Uterine Immune Profile May Help Women With Repeated Unexplained Embryo Implantation Failure After In Vitro Fertilization. Am. J. Reprod. Immunol..

[148] Abdolmohammadi-Vahid S., Danaii S., Hamdi K., Jadidi-Niaragh F., Ahmadi M., Yousefi M. (2016). Novel immunotherapeutic approaches for treatment of infertility. Biomed. Pharmacother..

[149] Nakagawa K., Kwak-Kim J., Ota K., Kuroda K., Hisano M., Sugiyama R., Yamaguchi K. (2015). Immunosuppression with tacrolimus improved reproductive outcome of women with repeated implantation failure and elevated peripheral blood TH1/TH2 cell ratios. Am. J. Reprod. Immunol..

[150] Nakagawa K., Kwak-Kim J., Kuroda K., Sugiyama R., Yamaguchi K. (2017). Immunosuppressive treatment using tacrolimus promotes pregnancy outcome in infertile women with repeated implantation failures. Am. J. Reprod. Immunol..

[151] Yamaguchi K. (2019). Tacrolimus treatment for infertility related to maternal-fetal immune interactions. Am. J. Reprod. Immunol..

[152] Ahmadi M., Abdolmohamadi-Vahid S., Ghaebi M., Dolati S., Abbaspour-Aghdam S., Danaii S., Berjis K., Madadi-Javid R., Nouri Z., Siahmansouri H. (2019). Sirolimus as a new drug to treat RIF patients with elevated Th17/Treg ratio: A double-blind, phase II randomized clinical trial. Int. Immunopharmacol..

[153] Yoshioka S., Fujiwara H., Nakayama T., Kosaka K., Mori T., Fujii S. (2006). Intrauterine administration of autologous peripheral blood mononuclear cells promotes implantation rates in patients with repeated failure of IVF-embryo transfer. Hum. Reprod..

[154] Makrigiannakis A., BenKhalifa M., Vrekoussis T., Mahjub S., Kalantaridou S.N., Gurgan T. (2015). Repeated implantation failure: A new potential treatment option. Eur. J. Clin. Investig..

[155] Achilli C., Duran-Retamal M., Saab W., Serhal P., Seshadri S. (2018). The role of immunotherapy in in vitro fertilization and recurrent pregnancy loss: A systematic review and meta-analysis. Fertil. Steril..

[156] Maleki-Hajiagha A., Razavi M., Rezaeinejad M., Rouholamin S., Almasi-Hashiani A., Pirjani R., Sepidarkish M. (2019). Intrauterine administration of autologous peripheral blood mononuclear cells in patients with recurrent implantation failure: A systematic review and meta-analysis. J. Reprod. Immunol..

[157] Yakin K., Oktem O., Urman B. (2019). Intrauterine administration of peripheral mononuclear cells in recurrent implantation failure: A systematic review and meta-analysis. Sci. Rep..

[158] Pourmoghadam Z., Abdolmohammadi-Vahid S., Pashazadeh F., Aghebati-Maleki L., Ansari F., Yousefi M. (2019). Efficacy of intrauterine administration of autologous peripheral blood mononuclear cells on the pregnancy outcomes in patients with recurrent implantation failure: A systematic review and meta-analysis. J. Reprod. Immunol..

